# Extracellular vesicles in cardiac repair and regeneration: Beyond stem-cell-based approaches

**DOI:** 10.3389/fcell.2022.996887

**Published:** 2022-09-02

**Authors:** Saveria Femminò, Filippo Bonelli, Maria Felice Brizzi

**Affiliations:** Department of Medical Sciences, University of Turin, Turin, Italy

**Keywords:** cardiac repair, cell therapy, extracellular vesicles, myocardial damage, inflammation

## Abstract

The adult human heart poorly regenerate after injury due to the low self-renewal capability retained by adult cardiomyocytes. In the last two decades, several clinical studies have reported the ability of stem cells to induce cardiac regeneration. However, low cell integration and survival into the tissue has limited stem-cell-based clinical approaches. More recently, the release of paracrine mediators including extracellular vesicles (EV) has been recognized as the most relevant mechanism driving benefits upon cell-based therapy. In particular, EV have emerged as key mediators of cardiac repair after damage, in terms of reduction of apoptosis, resolution of inflammation and new blood vessel formation. Herein, mechanisms involved in cardiac damage and regeneration, and current applications of EV and their small non-coding RNAs (miRNAs) in regenerative medicine are discussed.

## Introduction

According to the World Health Organization, cardiovascular diseases (CVDs) are the main cause of death worldwide, representing 31% of all global deaths. CVDs cover a wide range of disorders, including diseases of the cardiac muscle and vascular structures supplying oxygen to the heart, the brain, and other vital organs (Kaptoge et al., 2019). Inherited predisposition or long-lasting exposure to risk factors are considered the most relevant damaging inducers. Among them, heart tissue damaging takes on great importance, since the heart is largely a post-mitotic organ with limited regenerative capacity (Adamiak et al., 2018). Hence, after damage, cardiomyocyte death is a common endpoint, leading to the activation of the inflammatory process and resulting in the replacement of dead cells with fibrotic tissues (Thomas and Grisanti, 2020). Currently one of major medical challenges relies on the identification of novel approaches to limit the maladaptive changes in the shape and size messing up the normal electromechanical continuum of the ventricular muscle and compromising its contractility.

At this regard, several studies were aimed to enhance the heart regenerative potential. In particular, stem cells have been widely investigated as potential tool. Studies in animal models of ischemic cardiomyopathy suggest that stem cell transplantation independent of their origin can improve heart functional recovery after injury (Segers and Lee, 2008). The first clinical trials in patients generated encouraging results, showing benefits. However, stem cell paracrine action was reported as the most relevant and favorable mechanism of action (Gnecchi et al., 2005, 2008; Kupatt et al., 2005; Uemura et al., 2006; Boudoulas and Hatzopoulos, 2009). Stem cell-based therapy attenuates inflammation (van den Akker et al., 2013), reduces apoptosis of surrounding cells (Hobby et al., 2019), induces angiogenesis (Yong et al., 2018), and lessens the extent of fibrosis (Kudo et al., 2003). Nevertheless, evidence shows that despite treatment, cardiac regeneration is feeble. Therefore, to improve the engraftment, long-term survival and appropriate differentiation of transplanted stem cells within the cardiovascular tissue is still considered a clinical challenge.

Moreover, the invasive procedure that eventually fails to translate into heart tissue regeneration represents one of the most relevant hurdle associated with stem cell transplantation (Adamiak et al., 2018). Currently, extracellular vesicles (EV) are emerging as pivotal regulators in cell-based approaches (Riazifar et al., 2017). EV are a heterogeneous group (e.g., ectosomes, microparticles, microvesicles, exosomes and oncosomes) of fluid-filled spheres enclosed by a lipid bilayer. EV are released from all cell types, both in physiological and in pathological conditions and are involved in long-distance trafficking of their cargo. EV cargo senses the microenvironment and recapitulates protein, lipid and nucleic acid content commonly covered by their cell of origin (Shah et al., 2018). Thanks to these properties, EV are major drivers of intracellular communication and have been also considered valuable tools for biomarker discovery (Femminò et al., 2020). Evidence that EV released from stem-progenitor cells act as therapeutics mimicking their parental cell functions has indeed provided promises (Chimenti et al., 2010; Bobis-Wozowicz et al., 2015; Oszvald et al., 2020; Wang et al., 2020).

Since scar formation reflects the limited proliferative activity of cardiomyocytes, it has been suggested that modulation of cell cycle progression in cardiomyocyte may represent an alternative therapeutic option. During development, the heart structure depends on several growth factors mainly acting on the proliferating programs, while after birth, the heart size mostly relies on the hypertrophic growth rather than by cell proliferation (Ponnusamy et al., 2017). A complex network of proteins and transcription factors regulate the mitotic process, among them the cyclin dependent kinases (CDKs) and their required co-factors, the D-type Cyclins (Hassink et al., 2008; Ponnusamy et al., 2017). Previous studies successfully demonstrated that transgenic models expressing Cyclin D2 under the transcriptional regulation of the alpha-cardiac myosin heavy chain (MHC) promoter showed a better recovery after Myocardial Infarction (MI), with an increase in the number of living cardiomyocytes (Hassink et al., 2008). Moreover, phosphoinositide 3-kinase/protein kinase B (AKT), hippo-yes associate protein (YAP), and Wnt/β-catenin pathways have been found to contribute to cardiomyocyte proliferation (Ponnusamy et al., 2017).

The immune system exerts a strong influence on both repair and remodeling processes of the infarcted myocardium. Dying cardiomyocytes release a pool of signaling molecules that mobilize, recruit, and activate immune cells, triggering an inflammatory reaction (Chen and Frangogiannis, 2017). Specifically, neutrophils are attracted to the damaged area by CXC chemokines containing the ELR motif, such as CXCL8 and IL-8 (Kukielka et al., 1995a). Alternatively, monocytes’ and lymphocytes’ chemotaxis follow an increase in the secretion of CC chemokines like CCL2/MCP-1 (Dewald et al., 2005). Several studies have demonstrated that the type and the strength of the immune response can determine the extent of damage after cardiac injury (Frangogiannis, 2014). Indeed, as proinflammatory signaling is suppressed, macrophage subpopulations, mast cells, and lymphocytes activate the fibrogenic and angiogenic response, contributing to scar formation (Lai et al., 2019). Consequently, in chronic inflammatory conditions, a strong fibrogenic response occurs, resulting in hypertrophy and in the establishment of a wide scar. A critical role is played by immune cell subsets that participate in the suppression of the inflammatory response by secreting anti-inflammatory mediators, such as inteleukin-10 (IL-10) and transforming growth factor-β (TGF- β) (Lai et al., 2019). Targeting the inflammatory signals has been proposed as a potential pharmacological option in patients with MI, however, human heterogeneity, including age, gender, genetics, vascular damage, diabetes, and obesity, makes difficult the development of an appropriate therapeutic strategy (Huang and Frangogiannis, 2018).

The regulation of inflammation in cardiac remodelling is characterized by the damaged or dying cardiomyocytes that start to secret danger-associated molecular patterns (DAMPs). DAMPs bind to Pattern Recognition Receptors located on the cell surface of resident fibroblasts and other cell populations activating and inducing the production of cytokines such as interleukin-1 (IL-l), IL-2, interferon-y (IFN-y), and tumor necrosis factor- α (TNF-α). Regrettably, these molecules, which can promote cardiomyocyte death through the activation of specific molecular cascades, become cytotoxic for surrounding cells upon long-term exposure. As a result and without a proper anti-inflammatory response, they can activate a death chain reaction. Cytokine-mediated molecular mechanisms leading to cell death are discussed.

The pleiotropic cytokine tumor necrosis factor-α (TNF- α) acts by inducing receptor-mediated death in its target cells (VANEMPEL et al., 2005). TNF-Receptor (TNF-R) can induce both apoptotic and a necrotic cell death response. Fas, also called APO-1, is a member of this family which shares with TNF-R a common cytoplasmic death-signaling motif. Fas signaling has been well-characterized: it needs two molecules, FADD and FLICE, to induce signaling and to form functional complexes. FADD contains a cell death domain (D) in the C-terminus which is crucial to interact with Fas death domain (Tourneur and Chiocchia, 2010). The FADD N-terminus region contains a different motif, denoted as death effector domain (E), which is required for the binding to FLICE. FLICE and FADD interact *via* their respective death-effector domains. Interestingly, FLICE contains an interleukin-converting enzyme-like domain that may act as a driver of the cysteine protease cascade (Tourneur and Chiocchia, 2010). Both FADD and FLICE play a critical role in TNF-induced apoptosis.

The group of proinflammatory cytokines also includes IFN-y, IL-1, IL-2, IL-8 and the chemokine family. Both IL-2 and IL-1 (α and β) can induce the expression of TNF-α through a complex cascade (Hedayat et al., 2010). Furthermore, the stimulation of the apoptotic pathway may occur in response to nitric oxide production in cardiomyocytes, which in turn is induced by IL-1, IL-6, TNF-α and IFN-γ (Thomas et al., 2002; Umar and van der Laarse, 2010).

**FIGURE 1:**
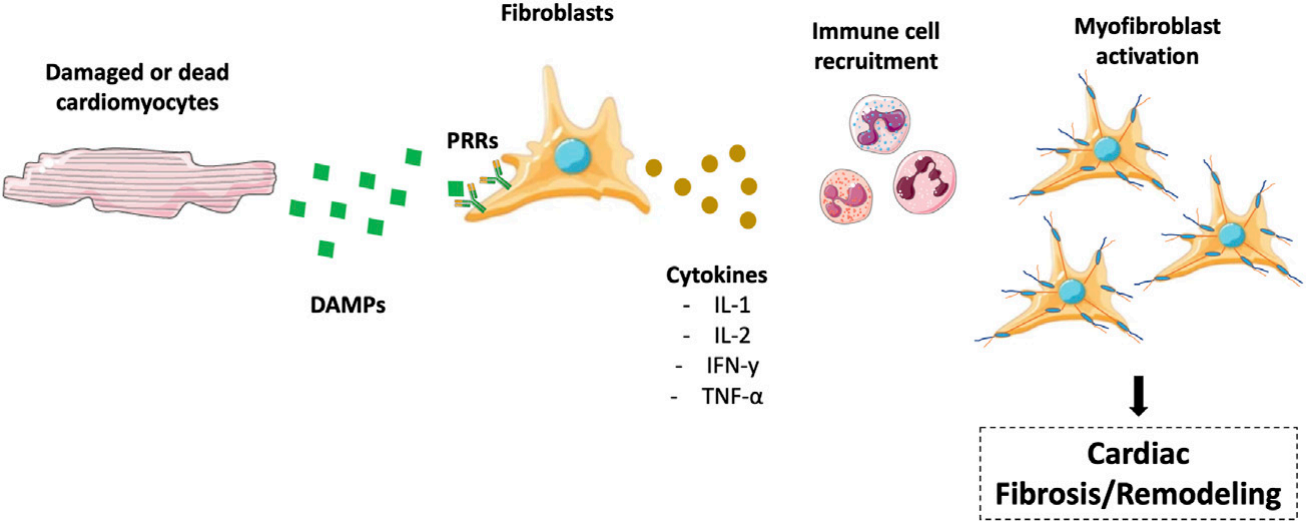
Regulation of inflammation in cardiac remodeling/fibrosis. Damaged or dead cardiomyocytes secrete DAMPs, which interact with PRRs to produce cytokines, including IL-l, IL-2, IFN-y, and TNF-α. These molecules promote immune cell recruitment in the heart, translating in myofibroblast activation and cardiac fibrosis. The figure was partly generated using Servier Medical Art templates, which are licensed under a Creative Commons Attribution 3.0 Unported License; https://smart.servier.com.

Therefore, after myocardial injury, a cytokine-enriched environment promotes immune cell recruitments and triggers the immune response (Figure 1). The resolution of inflammation will be discussed later.

In this review, mechanisms of cardiac damage and repair will be discussed. Particular attention will be devoted to discuss recent data on EV in cardiac regeneration.

## Mechanisms of cardiovascular repair and regeneration

Cardiac repair and regeneration involve several independent mechanisms. The processes that lead to cardiac regenerative responses include reduction of inflammation, cardiomyogenesis, and angiogenesis (Broughton et al., 2018).

### Reduction of inflammation

The progression towards a complete healing requires the resolution of the inflammatory process (Frangogiannis, 2012). Inflammation naturally occurs after injury and is required to set up regeneration and scar formation. Initially, the damaged myocardium causes an immune response regarded as debris and extracellular matrix (ECM) degradation (Lai et al., 2019). The second phase is represented by the recruitment and activation of fibroblasts leading to ECM deposition and angiogenesis (Frangogiannis, 2012, 2014). Mononuclear cell and mast cell responses are promoted by several molecules including TGF-β1, IL-8, histamine, TNF-α, IL-6, and ICAM-1 (Kukielka et al., 1995b). Monocytes, recruited into injured regions, differentiate into macrophages as the result of the effect of a hematopoietic growth factor, known as Macrophage Colony-Stimulating Factor (M-CSF) (Frangogiannis et al., 1998), while lymphocytes release IL-10, which suppresses inflammation through the inhibition of IL-6, IL-8, IL-12, TNF-α, IL-1α, and IL-1β secretion, and contribute to cardiac healing process (Frangogiannis et al., 2000). Fibroblasts accumulate within a week after infarction around the ischemic zone, and factors released in response to inflammation, such as VEGF, IL-8, and βFGF promote angiogenesis in the healing myocardium (Kukielka et al., 1995a). Additionally, neutrophil infiltration, *via* neutrophil–endothelial interactions, neutrophil rolling and activation of L-, E- and P-selectins, leukocyte β2 integrins, chemotaxis and chemokines, is one of the most relevant mechanisms involved in cell-mediated inflammatory response (Muller, 2002; Weil and Neelamegham, 2019). Although neutrophils should be committed towards cardiac repair, they can prolong damage in the injured myocardium. ICAM-1 activation in cardiac fibroblasts is recognized as a mechanism linked to neutrophil-mediated tissue damage. Indeed, ICAM-1 can be detected in ischemic areas approximately 3–6 h after injury and particularly in sites of neutrophil infiltration (Olivares-Silva et al., 2018).

### Cardiomyogenesis

New cardiomyocyte formation depends on resident cardiac stem cells (CSCs) and cardiac-derived progenitor cells (CPCs). CSCs mitotic activity is a rare event in the adult heart and the level of regeneration from resident cardiomyocytes is functionally trivial (Porrello and Olson, 2014). Pre-existing cardiomyocytes are the primary source of cardiomyocyte replacement after damage, however, several studies revealed an extremely limited expansion of cardiomyocytes from the pre-existing cardiomyocyte pool (Senyo et al., 2013; Torella et al., 2015). Cardiomyocyte turnover in humans declines with ageing, corresponding to1.9% in adolescent, 1% in the middle age, and 0.45% in the old age (Bergmann et al., 2009). Although the adult human heart has a limited cardiomyocyte regenerative capability, awareness of the mechanisms underlying cell renewal is crucial to develop strategies directed to cardiac recovery.

Lower vertebrates have a considerable plasticity to regenerate. In particular, it has been demonstrated a regenerative capacity of mouse heart after partial surgical resection, within the first week of postnatal life (Porrello et al., 2011). In adult murine cardiomyocytes, dedifferentiation and proliferation involve epigenomic reprogramming leading to downregulation of cardiac structure and functional genes and the activation of genes regulating cell cycle re-entry and proliferation (Zhang et al., 2015).

In several studies, using different species, CSCs have been extensively exploited as potential myocardial repair and regeneration cell source (Dawn et al., 2005; Linke et al., 2005; Tang et al., 2010; Bolli et al., 2013). In response to different environmental stimuli such as infarction injury, CSCs set out to divide, migrate, undergo lineage commitment, and mitigate cardiac injury (Leri et al., 2015). Bone marrow-derived cells (BMCs) are also considered a potential source of pro-regenerative cells (Kajstura et al., 2005; Janssens et al., 2006). In particular, BMCs influence resident cardiac cells to remodel the heart and improve the cardiac function through the release of several cytokines (Alfaro et al., 2010). Several clinical trials using BMCs to treat patients with heart failure have demonstrated an improvement of myocardial structure and function. However, the finding that only a few number of cells survive into the injured myocardium has represented the major drawback for their clinical application.

### Cell therapy

The post mitotic old paradigm regarding the heart has progressively became obsolete. Several evidence demonstrated that in the adult human heart a population of cardiac stem cells expressing stem cell markers, such as c-kit exists (60–63). This cell population can undergo cell division and replace dead cardiomyocytes; however, this process supports basal turnover to maintain tissue homeostasis, while appears inadequate to repair damaged areas (Weissman, 2000; Nadal-Ginard et al., 2003). Additionally, after MI or the development of heart failure, many cardiomyocytes, including progenitor cells, are lost, thereby, removed by macrophages. Therefore, dead heart areas undergo fibrosis, which results in a permanent impairment of the cardiac contractility.

In the last decades, stem cell transplantation has emerged as a new tool to boost regeneration process using a wide variety of potential stem/progenitor cell donors, that differ in their ability to survive, engraft, and differentiate (Wollert and Drexler, 2005). Pre-clinical studies in pig models using programmed cycles of ischemia/reperfusion (I/R) followed by the injection of embryonic Endothelial Progenitor Cells (eEPCs) demonstrated a marked reduction in the infarct size, through the activation of the phosphatidylinositol 3-kinase/AKT pathway (Kupatt et al., 2005). However, several evidence supports the theory that the beneficial effects of stem cell grafting are linked to the release of paracrine factors that modulate regeneration of damaged tissues (Gnecchi et al., 2005, 2008; Kupatt et al., 2005; Uemura et al., 2006; Boudoulas and Hatzopoulos, 2009). These factors, often enclosed in EV, are able to control several processes. Human mesenchymal stem cells (hMSCs) can be isolated from various sources, such as bone marrow, adipose tissue and umbilical cord (Wan Safwani et al., 2017; Choi et al., 2018). Mesenchymal stem cells (MSCs) display a strong immunosuppressor potential acting on CD4^+^ Th1, Th17, CD8^+^ T cells, and NK cells largely *via* the secretion of soluble factors including PGE2, IDO, HGF, and TGF-β1 (English et al., 2008; Xu et al., 2014). Moreover, it has been demonstrated that the immunosuppressive activity of MSCs is enhanced by IFN-γ stimulation (Klinker et al., 2017).

Stem cells also reduce apoptosis of surrounding cells. It has been demonstrated that intracardiac injection of cortical bone stem cells (CBSCs) in a swine pre-clinical model of I/R damage induces a significant reduction in the scar size, and accordingly, increases the pumping function (Hobby et al., 2019). CBSCs also increased the recruitment of macrophage and T-cells at day 7 of reperfusion, without altering the number of CD45 ^+^ cells (Hobby et al., 2019).

A different stem cell property supporting cardiac tissue regeneration relies on their proangiogenic capability. The loss of blood vessels and nutrients in the infarcted areas hamper the engraftment and the survival of new cardiomyocyte. hMSCs promote angiogenesis, enhance tissue repair and regeneration by the release soluble factors both in small and large animal models (Kuo et al., 2012; Hsiao et al., 2013; Tao et al., 2016). Evidence have been provided that hMSCs can downregulate leukocytes activation and functions during the development of atherosclerosis, demonstrating that MSCs mediate the repair of injured blood vessels (Yan et al., 2016). In addition, when blood vessels undergo permanent damage, hMSCs can support the regenerative process through the secretion of pro-angiogenic factors, such as vascular endothelial growth factor (VEGF) and by undergoing differentiation towards an endothelial cell phenotype.

Stem cell-based therapy also appears promising to lessen the extent of cardiac fibrosis and to prevent the progression towards heart failure. In the first phase, fibronectin is fold into the fibres, and secreted collagen along with other components form mature extracellular matrix (ECM), essential to promote scar formation. The attenuation of fibrosis seems to reflect the ability of different stem cell subpopulations to modulate ECM components rather than to directly replace and induce stem cell trans-differentiation. However, the exact mechanism(s) has not yet been determined and requires further investigation.

Several reviews (Fan et al., 2021; Lee et al., 2022; Mehanna et al., 2022) describe the role of different type of stem and progenitor cells in the restoration of the damaged heart. Nevertheless, evidence shows that only a few cardiac tissue undergoes regeneration. Therefore, to overcome these limitations, including poor engraftment, limited amelioration in cardiac function, and teratogenicity, EV from different sources have been explored as a novel approach. Herein, we will report the most relevant studies investigating EV-mediated cardiac repair.

## Extracellular vesicles and their role in cardiac regeneration

Extracellular vesicles (EV) have emerged as prognostic and therapeutic tools for several pathological conditions, including CVDs. As mentioned above, EV are a heterogeneous group of cell-derived membranous structures. According to the guidelines of the International Society for Extracellular Vesicles (ISEV), EV are defined based on their physical features, including size (small EV: <100 nm or <200 nm and medium/large EV: >200 nm) and density, and also on their cell origin, molecular markers, and function (Thery et al., 2018). Small EV include exosomes, which originate by a mechanism involving endosomal sorting complexes required for transport (ESCRT). Exosomes entail common components, such as the lipid bilayer (sphingomyelin, ceramides and cholesterol), transmembrane and internal proteins as Alix and TSG101, integrins, tetraspanins (CD63, CD81, and CD9), flotillin, and heat shock proteins (HSPs) (Skotland et al., 2019; Zhang et al., 2019). Moreover, exosomes also express specific components mirroring their cell of origin such as major histocompatibility complex (MHC) class-I and–II (Mashouri et al., 2019). ESCRT components are essential for exosome biogenesis. In fact, the loss of these proteins reduce exosome secretion in several cell types (Colombo et al., 2013). Furthermore, silencing the accessory ESCRT protein, Alix, increases the secretion of MHC class-II^+^ exosomes while reduces CD63 level. The observation that silencing Alix promotes the formation of medium/large vesicles, supports the notion that Alix strictly controls the nature/features of secreted vesicles (Colombo et al., 2013).

The formation of medium/large EV relies on the budding of plasma membrane (Doyle and Wang, 2019) and depends on signal-mediated intracellular calcium release, which in turn, triggers a cascade of biochemical and morphological changes in the phospholipid bilayer. Flippases, floppases and scramblases drive such modification by moving phosphatidylserine from the internal to the external side of the membrane. Calcium ions are also involved in the activation of proteolytic enzymes as calpains, which modify and disrupt the cytoskeleton, allowing vesiculation (Pollet et al., 2018).

In general, EV are secreted by all cell types and can be detected in many biological fluids, such as plasma, serum, saliva, urine (van Niel et al., 2018). In the last two decades, the ability of EV to influence target cell behaviour has gained particular interest. The effect of EV not only depends on their cell of origin but also on the microenvironment in which they have been released. Furthermore, the transfer of specific mRNAs or miRNAs to recipient cells relies on a targeted sorting mechanism. Specifically, miRNA sorting can be regulated by several types of RNA-binding proteins, such as heterogeneous nuclear ribonucleoproteins, argonaute 2, La protein, and Y-Box binding protein 1, which specifically bind and load miRNA into EV (Groot and Lee, 2020).

Since their cargo, consisting in proteins, lipids, amino acids, and RNAs, reflects their cell of origin, circulating EV have been proposed for biomarker discovery, and as prognostic and therapeutic tools. Moreover, based on the original observation that EV recapitulate the biological effect of their stem cell of origin (Hur et al., 2020), EV should be considered an alternative option to the cell-based therapy in cardiac regeneration. In addition, it has been widely demonstrated that treatments with EV secreted by stem or progenitor cells display substantial advantages compared to their cell of origin, such as lower immunogenicity, simple storage and production and more affordable cost compared to living stem cells. Thus, in recent years, the interest in EV as potential cell-free therapeutics, has rapidly expanded. The emerging role of EV in promoting cardiac regeneration will be discussed in the next paragraphs. Finally, compared to current exploitable biomarkers, EV unveil several advantages: i) non-invasive procedures can be used for their detection; ii) their cargo reflects disease progression and the response to treatment; iii) EV structure preserves their natural cargos during long-term storage.

### Extracellular vesicles and promotion of angiogenesis

After damage, new blood vessels formation is essential to rescue cardiac tissue. It has been demonstrated that EV released by CPCs have cardioprotective effects in the infarcted hearts by increasing blood vessel density (Barile et al., 2014). This effect relies on the enrichment of miR-132 in EV which improve neovessel formation by regulating its target RasGAP-p120 protein (Barile et al., 2014; Gallet et al., 2017). The pro-angiogenic effect of exosomes released by CPCs has been confirmed by Andriolo et al. (2018). CD31 expression was higher in cells exposed to CPCs-exosomes treatment. A recent study demonstrated that CPCs-derived exosomes promote angiogenesis by enhancing endothelial cell migration and, in particular it has been shown that CPCs cultured at 5% O_2_ generate exosomes with a greatest angiogenic potential (Dougherty et al., 2020). Moreover, it has been found that bioengineered CPCs-exosomes transfected with the pro-angiogenic miR-322 stimulate the angiogenic response in the damaged heart (Youn et al., 2019).

Exosomes obtained by MSCs pre-treated with atorvastatin increase arteriole and capillary density, improving cardiac function in the infarcted hearts (Huang et al., 2020). Therefore, it has been demonstrated that exosomes derived from MSCs exposed to ischemia, contain several proteins related to angiogenesis including platelet-derived growth factor (PDGF), epidermal growth factor (EGF) and fibroblast growth factor (FGF), inducing pro-angiogenic stimuli to promote tissue healing (Anderson et al., 2016).

It has been reported that the enrichment of miR-132 in MSC-exosomes promotes angiogenesis both *in-vitro* and *in-vivo* (Ma et al., 2018). Several evidence also identified adipose derived stem cells (ADSCs) as a relevant exosomes source involved in angiogenesis. ADSC-exosomes have been shown to prevent apoptosis and promote angiogenesis through the Wnt/β-catenin signaling pathway and miR-93-5p in the damaged heart (Cui et al., 2017; Liu et al., 2018). Exosomes derived from serum of patients with myocardial ischemia enhanced endothelial cell proliferation, migration and vessel formation. In a mouse hind-limb ischemia model, Li et al. (2018) demonstrated that ischemic exosomes significantly promoted blood flow recovery and enhanced neovascularization through miR-939-iNOS-NO pathway (Li et al., 2018). Similarly, it has been shown that adipose stem cell-derived EV are enriched in pro-angiogenic mRNAs able to rescue vascular and tissue damage in a hind-limb ischemia model (Figliolini et al., 2020).

### Extracellular vesicle and reduction of apoptosis

EV have been also explored for their anti-apoptotic effect during cardiac repair. MSC-exosomes alleviate cardiomyocyte apoptosis delaying the progression of cardiomyopathy, by decreasing the expression of pro-apoptotic protein Bax and increasing the expression of the pro-survival protein Bcl-2 (Sun et al., 2018). EV released by CPCs enriched in miRNAs miR-210, miR-132, and miR-146a-3p were found to reduce cardiomyocytes death by inhibiting the apoptotic process (Barile et al., 2014). In particular, miR-210 and miR-132 inhibit apoptosis in HL-1 cardiomyocyte cell line while miR-210-silencing significantly amplifies apoptosis. Downregulation of ephrin A3 and PTP1, two miR-210 targets, is associated with the anti-apoptotic effect. A different mechanism that contributes to protection against apoptosis is autophagy (Thorburn, 2008). Indeed, miR-30a transferred from exosomes, released by hypoxic cardiomyocytes, attenuates apoptosis by targeting beclin-1 and Atg12 genes (Yang et al., 2016). In addition, plasma exosomes reduce cell death after cardiac I/R injury. This effect relies on the cross-talk between the exosomal heat shock protein 70 and Toll-like receptor four and the activation of the extracellular signal-regulated protein kinases one and 2 (ERK1/2) and p38 mitogen-activated protein kinase (p38MAPK) (Vicencio et al., 2015). miR-199a-3p was found crucial for cardiac repair upon MI, both *ex-vivo* and an *in-vivo*. In particular, miR-199a-3p expression increases cardiomyocyte proliferation occurring in a damaged heart thereby improving the cardiac function (Eulalio et al., 2012). Taken together, these results indicate a beneficial effect of miR199a-3p in reducing the infarct size and preserving the cardiac function after MI. In a rat model of MI, Dergilev et al. (2020) tested the therapeutic potential of MSCs adapted to secrete the stem cell factor (SCF). Proteomic analysis revealed that these EV were enriched in chaperone and cytoskeleton proteins and in molecules associated with metabolic processes, which prevent harmful cardiac remodelling and confer improvement to the cardiac function. In an *ex-vivo* cardiac I/R model, endothelial cells-derived EV show cardioprotective properties. The enrichment of MEK1/2 and heat shock protein 90 (HSP90), a chaperone protein that stabilizes the folding and the heat stress of different proteins, in EV has been proposed for protection (Penna et al., 2020). Furthermore, endothelial cells-derived EV significantly increase the expression of the anti-apoptotic protein Bcl-2 in cardiomyocytes, suggesting a role in reducing cell death and conferring cardioprotection (Penna et al., 2020). A recent study demonstrated that EV isolated from serum of acute coronary syndrome (ACS) patients before percutaneous coronary intervention (PCI), display protection against I/R-induced damage in cardiomyocytes by activating the SAFE pathway (D’Ascenzo et al., 2021). EV cargo rearrangement was found crucial for the loss of protection of EV recovered from the same ACS patients after PCI (Femminò et al., 2021). Taken together, these studies provide evidence that EV, derived from different cell types, display anti-apoptotic properties, driving cardiac repair.

### Extracellular vesicle and resolution of inflammation

EV-mediated cardiac repair also relies on their effect on inflammation. Previous studies showed that EV derived from all cardiac cells regulate cytokine secretion and immune cell polarization, particularly M1 to M2 phenotype shift, through the interaction with infiltrating immune cells (Zhao et al., 2019; Lima Correa et al., 2021). EV secreted by cardiosphere-derived cells (CDCs), which obtained from biopsy of patient heart, were able to induce macrophage polarization *via* miR-181b (de Couto et al., 2017). Moreover, it has been demonstrated that a Y RNA fragment enriched in CDCs-EV modulates both IL-10 expression and secretion and improves cardiac repair (Cambier et al., 2017). More recently, it has been shown that EV derived from CPCs reduce the inflammatory process by modulating the expression of the pro-inflammatory cytokines, IL-1α, IL-2, and IL-6 (Lima Correa et al., 2021). Particularly, in the *in-vitro* model, CPCs-exosomes increased the number of anti-inflammatory M2 macrophages and reduced the number of pro-inflammatory monocytes and M1 macrophages. The observation that MSCs-exosomes injection in the infarcted heart mitigate inflammation by decreasing CD68 ^+^ macrophages as well as the enrichment of miR-24 in MSCs-exosomes and MSCs further supports the role of EV in solving the inflammatory state (Shao et al., 2017). Intravenous infusion of MSCs-exosomes in a mouse model of cardiomyopathy decreased circulating pro-inflammatory cytokines and regulated the balance between M1 and M2 macrophages through the activation JAK2/STAT6 signaling pathway (Sun et al., 2018). Consistently, Xu et al. (2019), found that MSCs-exosomes induce macrophage polarization towards M2 phenotype and the release of the anti-inflammatory cytokine IL-10, by M2 macrophages, by inhibiting NF-κB p65 nuclear translocation and AKT1/2 phosphorylation.

### Extracellular vesicle potential clinical application

**FIGURE 2:**
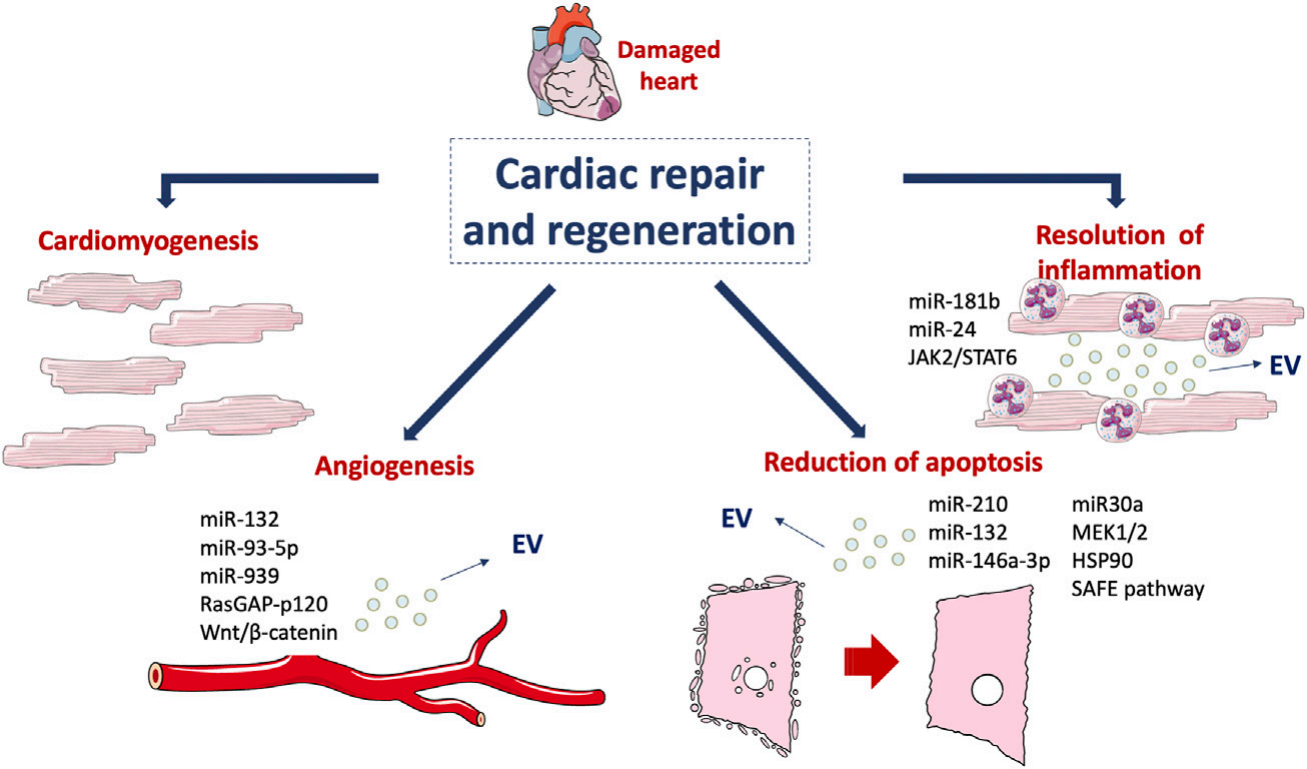
Mechanisms involved in cardiac regeneration. Cardiomyogenesis and EV-mediated effects, such as angiogenesis, reduction of apoptosis and resolution of inflammation drive the damaged cardiac tissue towards healing. This figure was partly generated using Servier Medical Art templates, which are licensed under a Creative Commons Attribution 3.0 Unported License; https://smart.servier.com.

In the last decade, several clinical trials provided evidence for EV as biomarkers of the increased risk of myocardial damage in CVDs. The prognostic potential of EV was reported using epicardial fat (eFat)-derived EV. The authors showed that these EV transfer profibrotic microRNA and proinflammatory cytokines in patients with atrial fibrillation (AF) (Shaihov-Teper et al., 2021). Similarly, it has been reported an association between exosomal microRNA profiling and adverse left ventricular remodelling (ALVR) after MI (Eyyupkoca et al., 2022). In particular, three microRNAs (miR-423-5p, miR-301a-3p and miR-374a-5p) were found differentially expressed in the follow-up period in patients with or without ALVR. Accordingly, circulating extracellular small non-coding RNAs (exRNAs) were found associated with inflammation and fibrosis in patients with ALVR (Danielson et al., 2018). The association between exRNAs and the ALVR phenotype after MI were proposed as biomarkers for the development of ALVR. The role of EV as prognostic and/or diagnostic biomarkers has been extensively evaluated. Indeed, in patients undergoing surgical aortic valve replacement (SAVR), the levels of circulating EV correlated with the left ventricle mass (LVM) regression and LDH release (Weber et al., 2020). In particular, lower levels of circulating EV were associated with an increased LVM and with higher LDH after SAVR, indicating that EV may be considered a prognostic predictor of patients’ clinical outcomes (Weber et al., 2020). In a recent study, it has been reported that circulating EV enriched in tissue factor are significantly higher in AF patients than in controls and have been correlated with the increased thrombotic risk of AF patients (Mørk et al., 2019). In a different clinical trial, higher levels of EV were found in ACS patients compared to stable angina patients undergoing PCI (Biasucci et al., 2012). These findings, besides confirming the potential application of EV as biomarkers of increased risk of myocardial damage, have provided evidence for their role as therapeutic targets in cardiovascular diseases. The most relevant effects of EV are summarized in Table 1 and represented in Figure 2.

**TABLE 1:** Summary of studies reporting EV-mediated effects in cardiac repair.

EV origin	Contents/Mediators	Effects	References
CPCs	miR-132; miR-210; miR146a-3p	Increase of blood vessel density; inhibition of apoptosis	Barile et al. (2014)
CPCs	n/a	Angiogenesis	Andriolo et al. (2018)
CPCs	n/a	Angiogenesis	Dougherty et al. (2020)
CPCs	miR-322	Angiogenesis	Youn et al. (2019)
MSCs	n/a	Increase of arteriole and capillary density	Huang et al. (2020)
MSCs	NF-kB; PDGF; EGF; FGF	Angiogenesis	Anderson et al. (2016)
MSCs	miR-132	Angiogenesis	Ma et al. (2018)
ADSCs	Wnt/β-catenin pathway; miR-93-5p	Angiogenesis; prevention of apoptosis	(Cui et al., 2017; Liu et al., 2018)
Serum of MI patients	miR-939-iNOS-NO pathway	Blood flow recovery; neovascularization	Li et al. (2018)
ADSCs	Neuregulin 1	Angiogenesis	Figliolini et al. (2020)
MSCs	#212121; JAK2-STAT6 pathway	Reduction of apoptosis; regulation of the balance of M1 and M2 macrophages	Sun et al. (2018)
Hypoxic cardiomyocytes	#212121; miR30a	Regulation of autophagy and apoptosis	Yang et al. (2016)
Plasma	#212121; ERK1/2; p38MAPK	Reduction of cell death	Vicencio et al. (2015)
Endothelial cells	#212121; MEK1/2; HSP90	Reduction of cell death	Penna et al. (2020)
Serum of ACS patients	#212121; SAFE pathway	Reduction of infarct size	D’Ascenzo et al. (2021)
CDCs	#212121; miR-181b	Macrophage polarization	de Couto et al. (2017)
CDCs	#212121; Y RNA fragment	Modulation of IL-10 expression	Cambier et al. (2017)
CPCs	#212121; n/a	Modulation of pro-inflammatory cytokines	Lima Correa et al. (2021)
MSCs	#212121; miR-24-3p	Reduction of pro-inflammatory monocytes	Shao et al. (2017)
MSCs	#212121; NF-kB p65; AKT1/2	Modulation of IL-10 expression	Xu et al. (2019)

## Conclusion

It has become even more evident that different mechanisms are involved in cardiac repair and regeneration. Cardiovascular diseases such as MI benefits from cell-based therapies mainly in small animal models, since several limitations, including poor engraftment, limited improvement in cardiac function, and teratogenicity, were recognized in human studies. In fact, cell-based therapies have demonstrated endogenous cardiomyocyte proliferation after MI mainly associated to progenitor and stem cell–derived factors. In particular, EV derived from CDCs, CPCs, MSCs, plasma, serum, play a central role in angiogenesis, resolution of inflammation and apoptosis of damaged heart. Moreover, since EV contain a large number of biologically active factors mainly recapitulating the microenvironment of their released cells, EV specific cargo has been recognized as promising biomarkers and therapeutic target in CVDs.

Finally, although different preclinical studies support the potential application of EV as cell-free approach, further data are required to ensure effectiveness and safety in humans. Moreover, protocols of standardization for dosing, quality control and scalable EV production are still missing for the clinical translation.

## References

[B1] Adamiak M. Cheng G. Bobis-Wozowicz S. Zhao L. Kedracka-Krok S. Samanta A. (2018). Induced Pluripotent Stem Cell (iPSC)-derived extracellular vesicles are safer and more effective for cardiac repair than iPSCs. Circ. Res. 122, 296–309. 10.1161/CIRCRESAHA.117.311769 29118058 PMC5775034

[B2] Alfaro M. P. Vincent A. Saraswati S. Thorne C. A. Hong C. C. Lee E. (2010). sFRP2 suppression of bone morphogenic protein (BMP) and Wnt signaling mediates mesenchymal stem cell (MSC) self-renewal promoting engraftment and myocardial repair. J. Biol. Chem. 285, 35645–35653. 10.1074/jbc.M110.135335 20826809 PMC2975189

[B3] Anderson J. D. Johansson H. J. Graham C. S. Vesterlund M. Pham M. T. Bramlett C. S. (2016). Comprehensive proteomic analysis of mesenchymal stem cell exosomes reveals modulation of angiogenesis via nuclear factor-KappaB signaling. Stem cells Dayt. Ohio) 34, 601–613. 10.1002/stem.2298 PMC5785927 26782178

[B4] Andriolo G. Provasi E. Lo Cicero V. Brambilla A. Soncin S. Torre T. (2018). Exosomes from human cardiac progenitor cells for therapeutic applications: Development of a GMP-grade manufacturing method. Front. Physiol. 9, 1169. 10.3389/fphys.2018.01169 30197601 PMC6117231

[B5] Barile L. Lionetti V. Cervio E. Matteucci M. Gherghiceanu M. Popescu L. M. (2014). Extracellular vesicles from human cardiac progenitor cells inhibit cardiomyocyte apoptosis and improve cardiac function after myocardial infarction. Cardiovasc. Res. 103, 530–541. 10.1093/cvr/cvu167 25016614

[B6] Bearzi C. Rota M. Hosoda T. Tillmanns J. Nascimbene A. De Angelis A. (2007). Human cardiac stem cells. Proc. Natl. Acad. Sci. U. S. A. 104, 14068. 10.1073/pnas.0706760104 17709737 PMC1955818

[B7] Beltrami A. P. Barlucchi L. Torella D. Baker M. Limana F. Chimenti S. (2003). Adult cardiac stem cells are multipotent and support myocardial regeneration. Cell 114, 763–776. 10.1016/S0092-8674(03)00687-1 14505575

[B8] Bergmann O. Bhardwaj R. D. Bernard S. Zdunek S. Barnabé-Heider F. Walsh S. (2009). Evidence for cardiomyocyte renewal in humans. Sci. (New York, N.Y.) 324, 98–102. 10.1126/science.1164680 PMC2991140 19342590

[B9] Biasucci L. M. Porto I. Di Vito L. De Maria G. L. Leone A. M. Tinelli G. (2012). Differences in microparticle release in patients with acute coronary syndrome and stable angina. Circ. J. 76, 2174–2182. 10.1253/circj.cj-12-0068 22664782

[B10] Bobis-Wozowicz S. Kmiotek K. Sekula M. Kedracka-Krok S. Kamycka E. Adamiak M. (2015). Human induced pluripotent stem cell-derived microvesicles transmit RNAs and proteins to recipient mature heart cells modulating cell fate and behavior. Stem Cells 33, 2748–2761. 10.1002/stem.2078 26031404

[B11] Bolli R. Tang X.-L. Sanganalmath S. K. Rimoldi O. Mosna F. Abdel-Latif A. (2013). Intracoronary delivery of autologous cardiac stem cells improves cardiac function in a porcine model of chronic ischemic cardiomyopathy. Circulation 128, 122–131. 10.1161/CIRCULATIONAHA.112.001075 23757309 PMC3807652

[B12] Boudoulas K. D. Hatzopoulos A. K. (2009). Cardiac repair and regeneration: The rubik’s cube of cell therapy for heart disease. Dis. Model. Mech. 2, 344–358. 10.1242/dmm.000240 19553696 PMC2707103

[B13] Broughton K. M. Wang B. J. Firouzi F. Khalafalla F. Dimmeler S. Fernandez-Aviles F. (2018). Mechanisms of cardiac repair and regeneration. Circ. Res. 122, 1151–1163. 10.1161/CIRCRESAHA.117.312586 29650632 PMC6191043

[B14] Cambier L. de Couto G. Ibrahim A. Echavez A. K. Valle J. Liu W. (2017). Y RNA fragment in extracellular vesicles confers cardioprotection via modulation of IL-10 expression and secretion. EMBO Mol. Med. 9, 337–352. 10.15252/emmm.201606924 28167565 PMC5331234

[B15] Chen B. Frangogiannis N. G. (2017). Immune cells in repair of the infarcted myocardium. Microcirculation 24, e12305. 10.1111/micc.12305 27542099

[B16] Chimenti I. Smith R. R. Li T. S. Gerstenblith G. Messina E. Giacomello A. (2010). Relative roles of direct regeneration versus paracrine effects of human cardiosphere-derived cells transplanted into infarcted mice. Circ. Res. 106, 971–980. 10.1161/CIRCRESAHA.109.210682 20110532 PMC4317351

[B17] Choi J. R. Yong K. W. Choi J. Y. (2018). Effects of mechanical loading on human mesenchymal stem cells for cartilage tissue engineering. J. Cell. Physiol. 233, 1913–1928. 10.1002/jcp.26018 28542924

[B18] Colombo M. Moita C. van Niel G. Kowal J. Vigneron J. Benaroch P. (2013). Analysis of ESCRT functions in exosome biogenesis, composition and secretion highlights the heterogeneity of extracellular vesicles. J. Cell Sci. 126, 5553–5565. 10.1242/jcs.128868 24105262

[B19] Cui X. He Z. Liang Z. Chen Z. Wang H. Zhang J. (2017). Exosomes from adipose-derived mesenchymal stem cells protect the myocardium against ischemia/reperfusion injury through wnt/β-catenin signaling pathway. J. Cardiovasc. Pharmacol. 70, 225–231. 10.1097/FJC.0000000000000507 28582278 PMC5642342

[B20] Danielson K. M. Shah R. Yeri A. Liu X. Camacho Garcia F. Silverman M. (2018). Plasma circulating extracellular RNAs in left ventricular remodeling post-myocardial infarction. EBioMedicine 32, 172–181. 10.1016/j.ebiom.2018.05.013 29779700 PMC6020713

[B21] D’Ascenzo F. Femminò S. Ravera F. Angelini F. Caccioppo A. Franchin L. (2021). Extracellular vesicles from patients with Acute Coronary Syndrome impact on ischemia-reperfusion injury. Pharmacol. Res. 170, 105715. 10.1016/j.phrs.2021.105715 34111564

[B22] Dawn B. Stein A. B. Urbanek K. Rota M. Whang B. Rastaldo R. (2005). Cardiac stem cells delivered intravascularly traverse the vessel barrier, regenerate infarcted myocardium, and improve cardiac function. Proc. Natl. Acad. Sci. U. S. A. 102, 3766–3771. 10.1073/pnas.0405957102 15734798 PMC553298

[B23] de Couto G. Gallet R. Cambier L. Jaghatspanyan E. Makkar N. Dawkins J. F. (2017). Exosomal MicroRNA transfer into macrophages mediates cellular postconditioning. Circulation 136, 200–214. 10.1161/CIRCULATIONAHA.116.024590 28411247 PMC5505791

[B24] Dergilev K. V. Shevchenko E. K. Tsokolaeva Z. I. Beloglazova I. B. Zubkova E. S. Boldyreva M. A. (2020). Cell sheet comprised of mesenchymal stromal cells overexpressing stem cell factor promotes epicardium activation and heart function improvement in a rat model of myocardium infarction. Int. J. Mol. Sci. 21, E9603. 10.3390/ijms21249603 33339427 PMC7766731

[B25] Dewald O. Zymek P. Winkelmann K. Koerting A. Ren G. Abou-Khamis T. (2005). CCL2/monocyte chemoattractant protein-1 regulates inflammatory responses critical to healing myocardial infarcts. Circ. Res. 96, 881–889. 10.1161/01.RES.0000163017.13772.3a 15774854

[B26] Dougherty J. A. Patel N. Kumar N. Rao S. G. Angelos M. G. Singh H. (2020). Human cardiac progenitor cells enhance exosome release and promote angiogenesis under physoxia. Front. Cell Dev. Biol. 8, 130. 10.3389/fcell.2020.00130 32211408 PMC7068154

[B27] Doyle L. M. Wang M. Z. (2019). Overview of extracellular vesicles, their origin, composition, purpose, and methods for exosome isolation and analysis. Cells 8, 724. 10.3390/cells8070727 PMC6678302 31311206

[B28] Ellison G. M. Vicinanza C. Smith A. J. Aquila I. Leone A. Waring C. D. (2013). Adult c-kitpos cardiac stem cells are necessary and sufficient for functional cardiac regeneration and repair. Cell 154, 827–842. 10.1016/j.cell.2013.07.039 23953114

[B29] English K. Barry F. P. Mahon B. P. (2008). Murine mesenchymal stem cells suppress dendritic cell migration, maturation and antigen presentation. Immunol. Lett. 115, 50–58. 10.1016/j.imlet.2007.10.002 18022251

[B30] Eulalio A. Mano M. Dal Ferro M. Zentilin L. Sinagra G. Zacchigna S. (2012). Functional screening identifies miRNAs inducing cardiac regeneration. Nature 492, 376–381. 10.1038/nature11739 23222520

[B31] Eyyupkoca F. Ercan K. Kiziltunc E. Ugurlu I. B. Kocak A. Eyerci N. (2022). Determination of microRNAs associated with adverse left ventricular remodeling after myocardial infarction. Mol. Cell. Biochem. 477, 781–791. 10.1007/s11010-021-04330-y 35048282

[B32] Fan D. Wu H. Pan K. Peng H. Wu R. (2021). Regenerating damaged myocardium: A review of stem-cell therapies for heart failure. Cells 10, 3125. 10.3390/cells10113125 34831347 PMC8625160

[B33] Femminò S. D’Ascenzo F. Ravera F. Comità S. Angelini F. Caccioppo A. (2021). Percutaneous coronary intervention (PCI) reprograms circulating extracellular vesicles from ACS patients impairing their cardio-protective properties. Int. J. Mol. Sci. 22, 10270. 10.3390/ijms221910270 34638611 PMC8508604

[B34] Femminò S. Penna C. Margarita S. Comità S. Brizzi M. F. Pagliaro P. (2020). Extracellular vesicles and cardiovascular system: Biomarkers and cardioprotective effectors. Vasc. Pharmacol. 135, 106790. 10.1016/j.vph.2020.106790 32861822

[B35] Figliolini F. Ranghino A. Grange C. Cedrino M. Tapparo M. Cavallari C. (2020). Extracellular vesicles from adipose stem cells prevent muscle damage and inflammation in a mouse model of hind limb ischemia: Role of neuregulin-1. Arterioscler. Thromb. Vasc. Biol. 40, 239–254. 10.1161/ATVBAHA.119.313506 31665908

[B36] Frangogiannis N. G. Mendoza L. H. Lindsey M. L. Ballantyne C. M. Michael L. H. Smith C. W. (2000). IL-10 is induced in the reperfused myocardium and may modulate the reaction to injury. J. Immunol. 165, 2798–2808. 10.4049/jimmunol.165.5.2798 10946312

[B37] Frangogiannis N. G. (2012). Regulation of the inflammatory response in cardiac repair. Circ. Res. 110, 159–173. 10.1161/CIRCRESAHA.111.243162 22223212 PMC3690135

[B38] Frangogiannis N. G. (2014). The immune system and the remodeling infarcted heart: Cell biological insights and therapeutic opportunities.. J. Cardiovasc. Pharmacol. 63, 185–195. 10.1097/FJC.0000000000000003 24072174 PMC3949163

[B39] Frangogiannis N. G. Youker K. A. Rossen R. D. Gwechenberger M. Lindsey M. H. Mendoza L. H. (1998). Cytokines and the microcirculation in ischemia and reperfusion. J. Mol. Cell. Cardiol. 30, 2567–2576. 10.1006/jmcc.1998.0829 9990529

[B40] Gallet R. Dawkins J. Valle J. Simsolo E. de Couto G. Middleton R. (2017). Exosomes secreted by cardiosphere-derived cells reduce scarring, attenuate adverse remodelling, and improve function in acute and chronic porcine myocardial infarction. Eur. Heart J. 38, 201–211. 10.1093/eurheartj/ehw240 28158410 PMC5837390

[B41] Gnecchi M. He H. Liang O. D. Melo L. G. Morello F. Mu H. (2005). Paracrine action accounts for marked protection of ischemic heart by Akt-modified mesenchymal stem cells. Nat. Med. 11, 367–368. 10.1038/nm0405-367 15812508

[B42] Gnecchi M. Zhang Z. Ni A. Dzau V. J. (2008). Paracrine mechanisms in adult stem cell signaling and therapy. Circ. Res. 103, 1204–1219. 10.1161/CIRCRESAHA.108.176826 19028920 PMC2667788

[B43] Groot M. Lee H. (2020). Sorting mechanisms for MicroRNAs into extracellular vesicles and their associated diseases. Cells 9, 1044. 10.3390/cells9041044 PMC7226101 32331346

[B44] Hassink R. J. Pasumarthi K. B. Nakajima H. Rubart M. Soonpaa M. H. De La Rivière A. B. (2008). Cardiomyocyte cell cycle activation improves cardiac function after myocardial infarction. Cardiovasc. Res. 78, 18–25. 10.1093/cvr/cvm101 18079102 PMC2653079

[B45] Hedayat M. Mahmoudi M. J. Rose N. R. Rezaei N. (2010). Proinflammatory cytokines in heart failure: Double-edged swords. Heart fail. Rev. 15, 543–562. 10.1007/s10741-010-9168-4 20405319

[B46] Hobby A. R. H. Sharp T. E. Berretta R. M. Borghetti G. Feldsott E. Mohsin S. (2019). Cortical bone-derived stem cell therapy reduces apoptosis after myocardial infarction. Am. J. Physiol. Heart Circ. Physiol. 317, H820–H829. 10.1152/ajpheart.00144.2019 31441690 PMC6843016

[B47] Hsiao S. T. Lokmic Z. Peshavariya H. Abberton K. M. Dusting G. J. Lim S. Y. (2013). Hypoxic conditioning enhances the angiogenic paracrine activity of human adipose-derived stem cells. Stem Cells Dev. 22, 1614–1623. 10.1089/scd.2012.0602 23282141 PMC3653395

[B48] Huang P. Wang L. Li Q. Tian X. Xu J. Xu J. (2020). Atorvastatin enhances the therapeutic efficacy of mesenchymal stem cells-derived exosomes in acute myocardial infarction via up-regulating long non-coding RNA H19. Cardiovasc. Res. 116, 353–367. 10.1093/cvr/cvz139 31119268 PMC8204482

[B49] Huang S. Frangogiannis N. G. (2018). Anti-inflammatory therapies in myocardial infarction: Failures, hopes and challenges. Br. J. Pharmacol. 175, 1377–1400. 10.1111/bph.14155 29394499 PMC5901181

[B50] Hur Y. H. Cerione R. A. Antonyak M. A. (2020). Extracellular vesicles and their roles in stem cell biology. Stem cells Dayt. Ohio) 38, 469–476. 10.1002/stem.3140 PMC7703835 31828924

[B51] Janssens S. Dubois C. Bogaert J. Theunissen K. Deroose C. Desmet W. (2006). Autologous bone marrow-derived stem-cell transfer in patients with ST-segment elevation myocardial infarction: Double-blind, randomised controlled trial. Lancet (London, Engl. 367, 113–121. 10.1016/S0140-6736(05)67861-0 16413875

[B52] Kajstura J. Rota M. Whang B. Cascapera S. Hosoda T. Bearzi C. (2005). Bone marrow cells differentiate in cardiac cell lineages after infarction independently of cell fusion. Circ. Res. 96, 127–137. 10.1161/01.RES.0000151843.79801.60 15569828

[B53] Kaptoge S. Pennells L. De Bacquer D. Cooney M. T. Kavousi M. Stevens G. (2019). World Health organization cardiovascular disease risk charts: Revised models to estimate risk in 21 global regions. Lancet. Glob. Health 7, e1332–e1345. 10.1016/S2214-109X(19)30318-3 31488387 PMC7025029

[B54] Klinker M. W. Marklein R. A. Lo Surdo J. L. Wei C.-H. Bauer S. R. (2017). Morphological features of IFN-γ–stimulated mesenchymal stromal cells predict overall immunosuppressive capacity. Proc. Natl. Acad. Sci. U. S. A. 114, E2598–E2607. 10.1073/pnas.1617933114 28283659 PMC5380055

[B55] Kudo M. Wang Y. Wani M. A. Xu M. Ayub A. Ashraf M. (2003). Implantation of bone marrow stem cells reduces the infarction and fibrosis in ischemic mouse heart. J. Mol. Cell. Cardiol. 35, 1113–1119. 10.1016/S0022-2828(03)00211-6 12967634

[B56] Kukielka G. L. Smith C. W. LaRosa G. J. Manning A. M. Mendoza L. H. Daly T. J. (1995a). Interleukin-8 gene induction in the myocardium after ischemia and reperfusion *in vivo* . J. Clin. Invest. 95, 89–103. 10.1172/JCI117680 7814650 PMC295378

[B57] Kukielka G. L. Smith C. W. Manning A. M. Youker K. A. Michael L. H. Entman M. L. (1995b). Induction of interleukin-6 synthesis in the myocardium. Potential role in postreperfusion inflammatory injury. Circulation 92, 1866–1875. 10.1161/01.cir.92.7.1866 7671371

[B58] Kuo Y.-R. Chen C.-C. Goto S. Lin P.-Y. Wei F.-C. Chen C.-L. (2012). Mesenchymal stem cells as immunomodulators in a vascularized composite allotransplantation. Clin. Dev. Immunol. 2012, 854846–854848. 10.1155/2012/854846 23227090 PMC3514826

[B59] Kupatt C. Kinkel R. Lamparter M. Von Brühl M. L. Pohl T. Horstkotte J. (2005). Retroinfusion of embryonic endothelial progenitor cells attenuates ischemia-reperfusion injury in pigs: Role of phosphatidylinositol 3-kinase/AKT kinase. Circulation 112, 117–122. 10.1161/CIRCULATIONAHA.104.524801 16159802

[B60] Lai S.-L. Marín-Juez R. Stainier D. Y. R. (2019). Immune responses in cardiac repair and regeneration: A comparative point of view. Cell. Mol. Life Sci. 76, 1365–1380. 10.1007/s00018-018-2995-5 30578442 PMC6420886

[B61] Lee C.-S. Kim J. Cho H.-J. Kim H.-S. (2022). Cardiovascular regeneration via stem cells and direct reprogramming: A review. Korean Circ. J. 52, 341–353. 10.4070/kcj.2022.0005 35502566 PMC9064703

[B62] Leri A. Rota M. Pasqualini F. S. Goichberg P. Anversa P. (2015). Origin of cardiomyocytes in the adult heart. Circ. Res. 116, 150–166. 10.1161/CIRCRESAHA.116.303595 25552694 PMC4283577

[B63] Li H. Liao Y. Gao L. Zhuang T. Huang Z. Zhu H. (2018). Coronary serum exosomes derived from patients with myocardial ischemia regulate angiogenesis through the miR-939-mediated nitric oxide signaling pathway. Theranostics 8, 2079–2093. 10.7150/thno.21895 29721064 PMC5928872

[B64] Lima Correa B. El Harane N. Gomez I. Rachid Hocine H. Vilar J. Desgres M. (2021). Extracellular vesicles from human cardiovascular progenitors trigger a reparative immune response in infarcted hearts. Cardiovasc. Res. 117, 292–307. 10.1093/cvr/cvaa028 32049348

[B65] Linke A. Müller P. Nurzynska D. Casarsa C. Torella D. Nascimbene A. (2005). Stem cells in the dog heart are self-renewing, clonogenic, and multipotent and regenerate infarcted myocardium, improving cardiac function. Proc. Natl. Acad. Sci. U. S. A. 102, 8966–8971. 10.1073/pnas.0502678102 15951423 PMC1157041

[B66] Liu J. Jiang M. Deng S. Lu J. Huang H. Zhang Y. (2018). miR-93-5p-Containing exosomes treatment attenuates acute myocardial infarction-induced myocardial damage. Mol. Ther. Nucleic Acids 11, 103–115. 10.1016/j.omtn.2018.01.010 29858047 PMC5852413

[B67] Ma T. Chen Y. Chen Y. Meng Q. Sun J. Shao L. (2018). MicroRNA-132, delivered by mesenchymal stem cell-derived exosomes, promote angiogenesis in myocardial infarction. Stem Cells Int. 2018, 3290372. 10.1155/2018/3290372 30271437 PMC6151206

[B68] Mashouri L. Yousefi H. Aref A. R. Ahadi A. M. Molaei F. Alahari S. K. (2019). Exosomes: Composition, biogenesis, and mechanisms in cancer metastasis and drug resistance. Mol. Cancer 18, 75. 10.1186/s12943-019-0991-5 30940145 PMC6444571

[B69] Mathur A. Martin J. (2004). Stem cells and repair of the heart. Lancet 364, 183–192. 10.1016/S0140-6736(04)16632-4 15246732

[B70] Mehanna R. A. Essawy M. M. Barkat M. A. Awaad A. K. Thabet E. H. Hamed H. A. (2022). Cardiac stem cells: Current knowledge and future prospects. World J. Stem Cells 14, 1–40. 10.4252/wjsc.v14.i1.1 35126826 PMC8788183

[B71] Mørk M. Andreasen J. J. Rasmussen L. H. Lip G. Y. H. Pedersen S. Bæk R. (2019). Elevated blood plasma levels of tissue factor-bearing extracellular vesicles in patients with atrial fibrillation. Thromb. Res. 173, 141–150. 10.1016/j.thromres.2018.11.026 30530119

[B72] Muller W. A. (2002). Leukocyte-endothelial cell interactions in the inflammatory response. Lab. Invest. 82, 521–533. 10.1038/labinvest.3780446 12003992

[B73] Nadal-Ginard B. Kajstura J. Leri A. Anversa P. (2003). Myocyte death, growth, and regeneration in cardiac hypertrophy and failure. Circulation Res. 92, 139–150. 10.1161/01.RES.0000053618.86362.DF 12574141

[B74] Olivares-Silva F. Landaeta R. Aránguiz P. Bolivar S. Humeres C. Anfossi R. (2018). Heparan sulfate potentiates leukocyte adhesion on cardiac fibroblast by enhancing Vcam-1 and Icam-1 expression. Biochim. Biophys. Acta. Mol. Basis Dis. 1864, 831–842. 10.1016/j.bbadis.2017.12.002 29222072

[B75] Oszvald Á. Szvicsek Z. Sándor G. O. Kelemen A. Soós A. Á. Pálóczi K. (2020). Extracellular vesicles transmit epithelial growth factor activity in the intestinal stem cell niche. Stem Cells 38, 291–300. 10.1002/stem.3113 31675158

[B76] Penna C. Femminò S. Tapparo M. Lopatina T. Fladmark K. E. Ravera F. (2020). The inflammatory cytokine IL-3 hampers cardioprotection mediated by endothelial cell-derived extracellular vesicles possibly via their protein cargo. Cells 10, 13. 10.3390/cells10010013 PMC7822476 33374685

[B77] Pollet H. Conrard L. Cloos A.-S. Tyteca D. (2018). Plasma membrane lipid domains as platforms for vesicle biogenesis and shedding? Biomolecules 8, E94. 10.3390/biom8030094 30223513 PMC6164003

[B78] Ponnusamy M. Li P.-F. Wang K. (2017). Understanding cardiomyocyte proliferation: An insight into cell cycle activity. Cell. Mol. Life Sci. 74, 1019–1034. 10.1007/s00018-016-2375-y 27695872 PMC11107761

[B79] Porrello E. R. Mahmoud A. I. Simpson E. Hill J. A. Richardson J. A. Olson E. N. (2011). Transient regenerative potential of the neonatal mouse heart. Sci. (New York, N.Y.) 331, 1078–1080. 10.1126/science.1200708 PMC3099478 21350179

[B80] Porrello E. R. Olson E. N. (2014). A neonatal blueprint for cardiac regeneration. Stem Cell Res. 13, 556–570. 10.1016/j.scr.2014.06.003 25108892 PMC4316722

[B81] Riazifar M. Pone E. J. Lötvall J. Zhao W. (2017). Stem cell extracellular vesicles: Extended messages of regeneration. Annu. Rev. Pharmacol. Toxicol. 57, 125–154. 10.1146/annurev-pharmtox-061616-030146 27814025 PMC5360275

[B82] Segers V. F. M. Lee R. T. (2008). Stem-cell therapy for cardiac disease. Nature 451, 937–942. 10.1038/nature06800 18288183

[B83] Senyo S. E. Steinhauser M. L. Pizzimenti C. L. Yang V. K. Cai L. Wang M. (2013). Mammalian heart renewal by pre-existing cardiomyocytes. Nature 493, 433–436. 10.1038/nature11682 23222518 PMC3548046

[B84] Shah R. Patel T. Freedman J. E. (2018). Circulating extracellular vesicles in human disease. N. Engl. J. Med. 379, 958–966. 10.1056/nejmra1704286 30184457

[B85] Shaihov-Teper O. Ram E. Ballan N. Brzezinski R. Y. Naftali-Shani N. Masoud R. (2021). Extracellular vesicles from epicardial fat facilitate atrial fibrillation. Circulation 143, 2475–2493. 10.1161/CIRCULATIONAHA.120.052009 33793321

[B86] Shao L. Zhang Y. Lan B. Wang J. Zhang Z. Zhang L. (2017). MiRNA-sequence indicates that mesenchymal stem cells and exosomes have similar mechanism to enhance cardiac repair. Biomed. Res. Int. 2017, 4150705. 10.1155/2017/4150705 28203568 PMC5292186

[B87] Skotland T. Hessvik N. P. Sandvig K. Llorente A. (2019). Exosomal lipid composition and the role of ether lipids and phosphoinositides in exosome biology. J. Lipid Res. 60, 9–18. 10.1194/jlr.R084343 30076207 PMC6314266

[B88] Sun X. Shan A. Wei Z. Xu B. (2018). Intravenous mesenchymal stem cell-derived exosomes ameliorate myocardial inflammation in the dilated cardiomyopathy. Biochem. Biophys. Res. Commun. 503, 2611–2618. 10.1016/j.bbrc.2018.08.012 30126637

[B89] Tang X.-L. Rokosh G. Sanganalmath S. K. Yuan F. Sato H. Mu J. (2010). Intracoronary administration of cardiac progenitor cells alleviates left ventricular dysfunction in rats with a 30-day-old infarction. Circulation 121, 293–305. 10.1161/CIRCULATIONAHA.109.871905 20048209 PMC2814341

[B90] Tao H. Han Z. Han Z. C. Li Z. (2016). Proangiogenic features of mesenchymal stem cells and their therapeutic applications. Stem Cells Int. 2016, 1314709. 10.1155/2016/1314709 26880933 PMC4736816

[B91] Thery C. Witwer K. W. Aikawa E. Acaraz M. J. Anderson Johnathon D. Andriantsitohaina R. (2018). Minimal information for studies of extracellular vesicles 2018 (MISEV2018): A position statement of the international society for extracellular vesicles and update of the MISEV2014 guidelines. J. Extracell. Vesicles 7, 1535750. 10.1080/20013078.2018.1535750 30637094 PMC6322352

[B92] Thomas H. E. Darwiche R. Corbett J. A. Kay T. W. H. (2002). Interleukin-1 plus γ-interferon-induced pancreatic β-cell dysfunction is mediated by β-cell nitric oxide production. Diabetes 51, 311–316. 10.2337/diabetes.51.2.311 11812737

[B93] Thomas T. P. Grisanti L. A. (2020). The dynamic interplay between cardiac inflammation and fibrosis. Front. Physiol. 11, 529075. 10.3389/fphys.2020.529075 33041853 PMC7522448

[B94] Thorburn A. (2008). Apoptosis and autophagy: Regulatory connections between two supposedly different processes. Apoptosis 13, 1–9. 10.1007/s10495-007-0154-9 17990121 PMC2601595

[B95] Torella D. Indolfi C. Nadal-Ginard B. (2015). Generation of new cardiomyocytes after injury: De novo formation from resident progenitors vs. replication of pre-existing cardiomyocytes. Ann. Transl. Med. 3, S8. 10.3978/j.issn.2305-5839.2015.02.17 26046095 PMC4437964

[B96] Tourneur L. Chiocchia G. (2010). Fadd: A regulator of life and death. Trends Immunol. 31, 260–269. 10.1016/j.it.2010.05.005 20576468

[B97] Uemura R. Xu M. Ahmad N. Ashraf M. (2006). Bone marrow stem cells prevent left ventricular remodeling of ischemic heart through paracrine signaling. Circ. Res. 98, 1414–1421. 10.1161/01.RES.0000225952.61196.39 16690882

[B98] Umar S. van der Laarse A. (2010). Nitric oxide and nitric oxide synthase isoforms in the normal, hypertrophic, and failing heart. Mol. Cell. Biochem. 333, 191–201. 10.1007/s11010-009-0219-x 19618122

[B99] van den Akker F. Deddens J. C. Doevendans P. A. Sluijter J. P. G. (2013). Cardiac stem cell therapy to modulate inflammation upon myocardial infarction. Biochim. Biophys. Acta 1830, 2449–2458. 10.1016/j.bbagen.2012.08.026 22975401

[B100] van Niel G. D’Angelo G. Raposo G. (2018). Shedding light on the cell biology of extracellular vesicles. Nat. Rev. Mol. Cell Biol. 19, 213–228. 10.1038/nrm.2017.125 29339798

[B101] Vanempel V. Bertrand A. Hofstra L. Crijns H. Doevendans P. Dewindt L. (2005). Myocyte apoptosis in heart failure. Cardiovasc. Res. 67, 21–29. 10.1016/j.cardiores.2005.04.012 15896727

[B102] Vicencio J. M. Yellon D. M. Sivaraman V. Das D. Boi-Doku C. Arjun S. (2015). Plasma exosomes protect the myocardium from ischemia-reperfusion injury. J. Am. Coll. Cardiol. 65, 1525–1536. 10.1016/j.jacc.2015.02.026 25881934

[B103] Wan Safwani W. K. Z. Choi J. R. Yong K. W. Ting I. Mat Adenan N. A. Pingguan-Murphy B. (2017). Hypoxia enhances the viability, growth and chondrogenic potential of cryopreserved human adipose-derived stem cells. Cryobiology 75, 91–99. 10.1016/j.cryobiol.2017.01.006 28108309

[B104] Wang Y. Han B. Wang Y. Wang C. Zhang H. Xue J. (2020). Mesenchymal stem cell–secreted extracellular vesicles carrying TGF-β1 up-regulate miR-132 and promote mouse M2 macrophage polarization. J. Cell. Mol. Med. 24, 12750–12764. 10.1111/jcmm.15860 32965772 PMC7686990

[B105] Weber A. Liu S. S. Cardone L. Rellecke P. Sixt S. U. Lichtenberg A. (2020). The course of circulating small extracellular vesicles in patients undergoing surgical aortic valve replacement. Biomed. Res. Int. 2020, 6381396. 10.1155/2020/6381396 32382562 PMC7193280

[B106] Weil B. R. Neelamegham S. (2019). Selectins and immune cells in acute myocardial infarction and post-infarction ventricular remodeling: Pathophysiology and novel treatments. Front. Immunol. 10, 300. 10.3389/fimmu.2019.00300 30873166 PMC6400985

[B107] Weissman I. L. (2000). Translating stem and progenitor cell biology to the clinic: Barriers and opportunities. Science 287, 1442–1446. 10.1126/science.287.5457.1442 10688785

[B108] Wollert K. C. Drexler H. (2005). Clinical applications of stem cells for the heart. Circ. Res. 96, 151–163. 10.1161/01.RES.0000155333.69009.63 15692093

[B109] Xu C. Yu P. Han X. Du L. Gan J. Wang Y. (2014). TGF-Β promotes immune responses in the presence of mesenchymal stem cells. J. Immunol. 192, 103–109. 10.4049/jimmunol.1302164 24293629

[B110] Xu R. Zhang F. Chai R. Zhou W. Hu M. Liu B. (2019). Exosomes derived from pro-inflammatory bone marrow-derived mesenchymal stem cells reduce inflammation and myocardial injury via mediating macrophage polarization. J. Cell. Mol. Med. 23, 7617–7631. 10.1111/jcmm.14635 31557396 PMC6815833

[B111] Yan T. Venkat P. Chopp M. Zacharek A. Ning R. Roberts C. (2016). Neurorestorative responses to delayed human mesenchymal stromal cells treatment of stroke in type 2 diabetic rats. Stroke 47, 2850–2858. 10.1161/STROKEAHA.116.014686 27729575 PMC5134897

[B112] Yang Y. Li Y. Chen X. Cheng X. Liao Y. Yu X. (2016). Exosomal transfer of miR-30a between cardiomyocytes regulates autophagy after hypoxia. J. Mol. Med. 94, 711–724. 10.1007/s00109-016-1387-2 26857375

[B113] Yong K. W. Choi J. R. Mohammadi M. Mitha A. P. Sanati-Nezhad A. Sen A. (2018). Mesenchymal stem cell therapy for ischemic tissues. Stem Cells Int. 2018, 8179075–8179111. 10.1155/2018/8179075 30402112 PMC6196793

[B114] Youn S.-W. Li Y. Kim Y.-M. Sudhahar V. Abdelsaid K. Kim H. W. (2019). Modification of cardiac progenitor cell-derived exosomes by miR-322 provides protection against myocardial infarction through nox2-dependent angiogenesis. Antioxidants (Basel, Switz. 8, 18. 10.3390/antiox8010018 PMC6356993 30634641

[B115] Zhang Y. Liu Y. Liu H. Tang W. H. (2019). Exosomes: Biogenesis, biologic function and clinical potential. Cell Biosci. 9, 19. 10.1186/s13578-019-0282-2 30815248 PMC6377728

[B116] Zhang Y. Zhong J. F. Qiu H. MacLellan W. R. Marbán E. Wang C. (2015). Epigenomic reprogramming of adult cardiomyocyte-derived cardiac progenitor cells. Sci. Rep. 5, 17686. 10.1038/srep17686 26657817 PMC4677315

[B117] Zhao J. Li X. Hu J. Chen F. Qiao S. Sun X. (2019). Mesenchymal stromal cell-derived exosomes attenuate myocardial ischaemia-reperfusion injury through miR-182-regulated macrophage polarization. Cardiovasc. Res. 115, 1205–1216. 10.1093/cvr/cvz040 30753344 PMC6529919

